# Metabolic Syndrome-Associated Erectile Dysfunction: Multiple Vascular Endothelial Dysfunction Mechanisms and Potential Therapeutic Targets

**DOI:** 10.7150/ijbs.120980

**Published:** 2025-09-12

**Authors:** Hao Wang, Jun Guo, Eric Chung

**Affiliations:** 1 Department of Andrology, Xiyuan Hospital, China Academy of Chinese Medical Sciences, Beijing, China.; 2 Guo Jun Qihuang Scholar Inheritance Studio, Ningxia Hui Autonomous Region Hospital of Traditional Chinese Medicine, Ningxia Hui Autonomous Region Academy of Traditional Chinese Medicine, Ningxia Hui Autonomous Region, China.; 3 Department of Urology, Princess Alexandra Hospital, University of Queensland, Brisbane, QLD, Australia.; 4 AndroUrology Centre, Brisbane, QLD, Australia.

**Keywords:** metabolic syndrome, erectile dysfunction, endothelial dysfunction, diabetes, hypertension

## Abstract

Metabolic syndrome (MetS) causes vascular structural abnormalities, nerve damage, hormonal level changes and other lesions, which promote the occurrence and development of erectile dysfunction (ED). Penile vascular endothelial dysfunction is an important pathological feature of MetS-associated ED, and has received increasing attention in recent years. MetS negatively affects penile cavernous vascular function through the synergistic effects of insulin resistance, dyslipidemia, hypertension and obesity. The multiple pathological process may lead to impaired endothelium-dependent vasodilation, progressive fibrosis and reduced penile vascular blood flow reserve. This review summarized several common mechanisms of penile vascular endothelial dysfunction in MetS-associated ED, deeply discussed the roles of common pathological manifestations of MetS such as glucose metabolism disorder, hypertension, dyslipidemia and obesity on penile vascular endothelium, and explored treatments targeting these mechanisms in order to provide potential therapeutic targets and strategies in patients with MetS-associated ED.

## 1. Introduction

Erectile dysfunction (ED) refers to the inability to achieve and/or maintain a sufficient erection to have satisfactory sexual life. It is not life-threatening, but can cause sexual life disorders and seriously affect the quality of relationships between patients and their sexual partners 1. It has been reported that the overall global prevalence was 13.1-71.2% 2, From a global study, 65% of men were not very satisfied with their erection hardness 3. Due to the differences in the regions, races, ages and cultural backgrounds of the study populations, the prevalence rates vary. The causes of ED mainly fall into three categories: psychological, organic and mixed. Most organic ED patients have vascular ED caused by the hemodynamic disorder, which is associated with endothelial dysfunction, arterial insufficiency, and/or venous occlusive dysfunction 4. ED is an early warning sign of cardiovascular disease 5, and it is also strongly associated with metabolic diseases 6. A number of metabolic disorders, including dyslipidemia, glucose intolerance, insulin resistance, and obesity, play important roles in the induction of ED, which is particularly prevalent in many middle-aged and elderly patients 7.

Metabolic syndrome (MetS) is a cluster of metabolic abnormalities that starts with insulin resistance and continues with the abdominal obesity, diabetes mellitus (DM), dyslipidemia, and hypertension 8. In United States, it affects approximately 35-39% of the population 9, and according to the data of the International Diabetes Federation, the prevalence of MetS was highest in the Eastern Mediterranean Region (36.6%) and lowest in the Africa (23.1%) 10. MetS can increase the risk of a number of diseases and adverse events, with ED being relatively prominent in male reproductive health 11. The Massachusetts Male Aging Study revealed an evolution of ED developing concurrently with the development of the Mets 12. Although many clinical trials suggested that patients with MetS have more propensity for ED, it is difficult for these cross-sectional studies or meta-analysis to elucidate how MetS induce ED. Recently, an Indian population study showed that ED prevalence rose with more MetS components, and flow-mediated dilation was significantly lower in ED patients (5.1 ± 1.1%) vs. non-ED (10.9 ± 3.3%) 13. Another study compared 100 men with MetS to an age- and body mass index-matched control group, results showed that the MetS group had an increased prevalence of ED (26.7% vs 13%), and a sixfold reduction in endothelial function score 14. Endothelial dysfunction may be the key mechanism in the pathogenesis of MetS-associated ED, and focusing on vascular endothelial function in the corpus cavernosum (CC) of MetS-associated ED holds significant promise for the discovery of drug targets and addressing the therapeutic challenges of organic ED. Previous outdated literature reviews did not focus on penile vascular endothelial function 15,16, or only focused one manifestation of MetS 17. Therefore, it is still necessary to conduct a comprehensive and in-depth review to discuss the penis vascular endothelial damage related to MetS, and provide reasonable suggestions for the discovery and development of drug targets through summarizing the relevant mechanisms 18-20.

## 2. Metabolic Syndrome-Induced Erectile Dysfunction: Penile Vascular Damage

Studies have highlighted the increasing importance of MetS, which is characterized by central obesity, impaired glucose metabolism, hypertriglyceridemia, low high-density lipoprotein cholesterol (HDL-C), and hypertension, in the pathogenesis and exacerbation of male reproductive issues 21,22, particularly in sexual dysfunction 23. MetS and male sexual dysfunction can occur independently but are often interconnected. Demir et al investigated the relationship between MetS and ED, and they found 74% of the 89 MetS patients had ED 24. Also, the severity of ED was directly correlated with MetS 25. Gamidov et al divided ED patients into Mets group and no Mets group, and results showed that ED appeared earlier in the Mets group (43.46 ± 9.87 vs. 50.38 ± 13.35 years, *p* < 0.05) and lasted longer (6.36 ± 4.13 vs. 3.55 ± 3.27 years, *p* < 0.05) 26. Also, half of the patients in the MetS group had severe ED, while severe ED was two times less common in the no MetS group 26. In fact, the mechanisms involved in MetS-associated ED are multifaceted 27. In MetS, ED was mainly arteriogenic, with 36.36% of cases showing subnormal androgen levels and 42.2% exhibiting neurogenic disorders 26. Arteriogenic ED patients presented penile hemodynamics alterations, with mean peak systolic velocity significantly lower comparatively to no MetS patients 28. Koca et al also used the penile color Doppler ultrasonography to detected the penile vascular condition in ED patients with or without Mets, and they found that there was a significant correlation between veno-occlusive dysfunction and Mets (left end-diastolic velocity 3.7 ± 3.3 cm/s vs. 2.7 ± 3.1 cm/s, *p* = 0.014; right end-diastolic velocity 3.8 ± 3.4 cm/s vs. 2.8 ± 3.3 cm/s, *p* = 0.016) 29.

## 3. Metabolic Syndrome-Induced Penile Vascular Damage: Endothelial Dysfunction

Erection is triggered by the stimulation of efferent autonomic nerves through either local sensory input or central psychogenic signals. This leads to the expansion of cavernosal and helicine arteries, enhancing blood flow into the lacunar spaces, alongside nitric oxide (NO)-induced relaxation of trabecular smooth muscle. The vascular endothelial function of the penis plays a crucial role in facilitating this process, and the vascular endothelium, a single-cell layer lining blood vessels, also responds to both biochemical and mechanical signals by generating various mediators 30. Endothelial cells produce various factors that regulate cellular adhesion, smooth muscle reactivity, proliferation, inflammation, and atherogenesis. Endothelial cells in a quiescent state promote anticoagulation, resist cellular adhesion, and facilitate vasodilation, whereas activated endothelial cells adopt procoagulant, adhesive, and vasoconstrictive functions. Degradation of endothelial integrity results in functional impairment, including disrupted NO secretion, reduced antiatherogenic effects, etc, and it also plays a role in organic ED.

**Figure 1:**
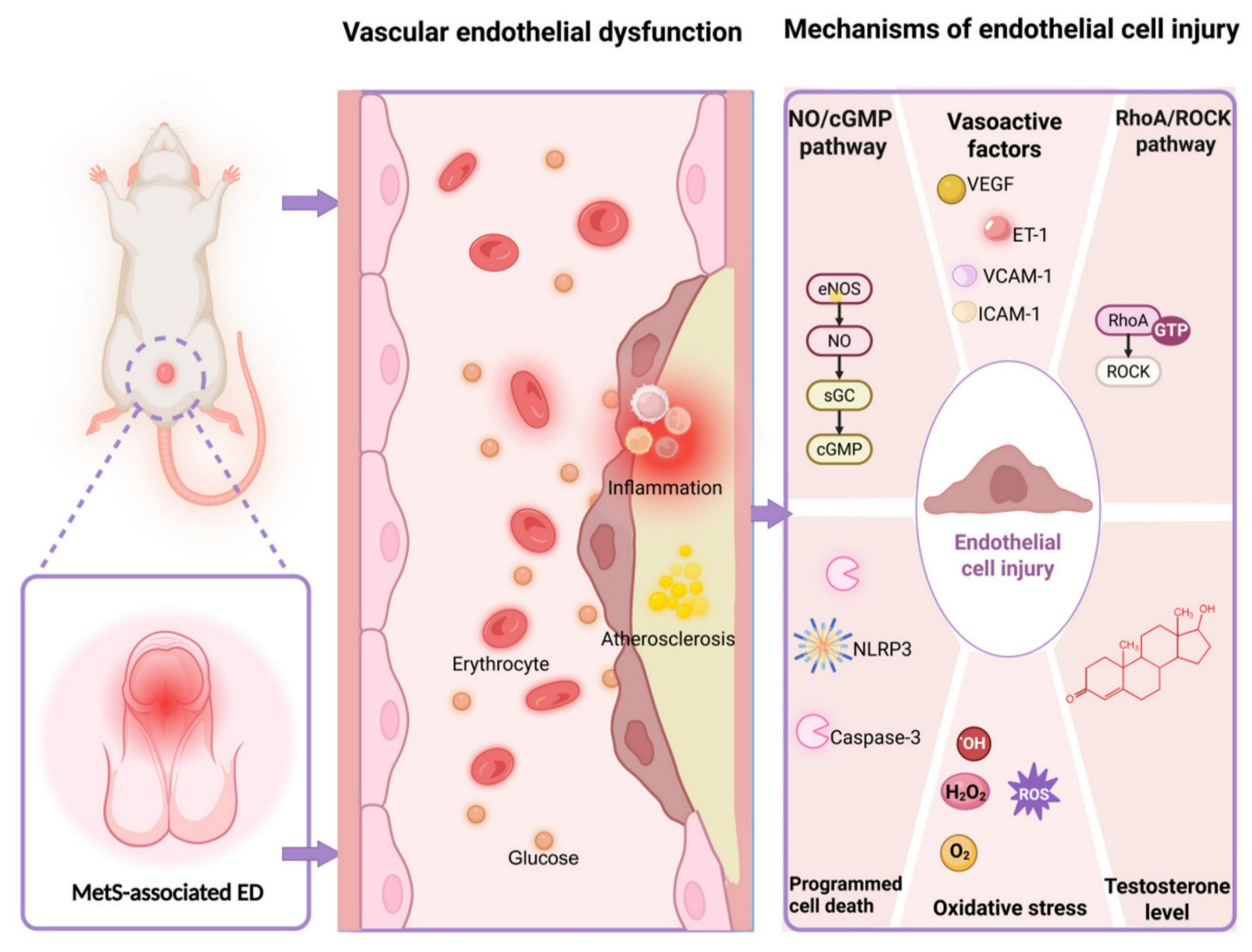
** Multiple vascular endothelial dysfunction mechanisms in MetS-associated ED.** Abbreviations: MetS, metabolic syndrome; ED, erectile dysfunction; NO, nitric oxide; cGMP, cyclic guanosine monophosphate; eNOS, endothelial nitric oxide synthase; sGC, soluble guanylate cyclase; VEGF, vascular endothelial growth factor; ET-1, endothelin-1; VCAM-1, vascular cell adhesion molecule-1; ICAM-1, intercellular cell adhesion molecule-1; RhoA, ras homolog family member A; ROCK, rho-associated coiled-coil containing protein kinase; GTP, guanosine triphosphatase; ROS, reactive oxygen species. Created in BioRender. Wang, H. (2025) https://BioRender.com/mcwyvzs.

MetS exerts its detrimental effects on CC vascular function through the synergistic interplay of insulin resistance, dyslipidemia, hypertension, and obesity 31. This multifaceted pathogenesis can result in impaired endothelium-dependent vasodilation, progressive fibrosis, and diminished blood flow reserve within the penile vasculature. While the specific mechanisms underlying endothelial dysfunction in CC may vary among the different pathological characteristics of MetS, but they converge on several common pathological pathways such as disturbances in both of the NO/Cyclic Guanosine Monophosphate (cGMP) and Ras homolog family member A (RhoA)/ Rho-associated coiled-coil containing protein kinase (ROCK) signaling pathways, imbalance of endothelium-derived vasoactive factors, alterations in sex hormone regulation, oxidative stress, and programmed cell death (Figure 1).

### 3.1 Nitric oxide/cyclic guanosine monophosphate pathway

When sexual stimulation activates the CC, the non-adrenergic non-cholinergic nerve endings and L-arginine within the endothelial cells can promote the release of NO from nitric oxide synthase (NOS) (such as endothelial NOS and neuronal NOS) 32. NO diffuses to the CC smooth muscle cells, activating soluble guanylate cyclase, which catalyzes the generation of guanosine triphosphate to produce the second messenger, cGMP. cGMP reduces intracellular calcium concentration via protein kinase G, leading to smooth muscle relaxation while compressing the veins to block reflux and maintain erection. We have exhaustively elucidated the mechanisms of NO/cGMP-mediated ED and the multiple pathways that cause penile vascular endothelial dysfunction by affecting NO/cGMP 33. NO is the main vasorelaxant released from the endothelial lining of the sinusoids and important associated vessels such as the cavernosal and helicine arteries. The perivascular smooth muscle relaxation dependence of the NO produced by endothelial NOS is regulated by insulin. Insulin-resistant condition contributes to factor the decreased availability of NO in the endothelium, and it may be caused by reductions in the enzyme endothelial NOS, a lack of substrate or cofactors for endothelial NOS, and alterations in intracellular signaling 34. NOS uncoupling not only results in reduced synthesis of NO, but enhances the capability of the enzyme to produce reactive oxygen species (ROS), and finally to be an important cause of MetS-associated ED and some other cardiovascular diseases 35.

### 3.2 Ras homolog family member A/Rho-associated coiled-coil containing protein kinase pathway

RhoA/ROCK pathway plays a significant role in MetS-associated ED. In addition to reducing NO release and impairing vasodilation, RhoA/ROCK pathway regulates the vascular endothelium of the CC by modulating endothelial cell contraction and disrupting endothelial barrier function. Activated RhoA enhances ROCK activity, promoting phosphorylation of myosin light chain and increasing vascular tension 36. ROCK regulates actin cytoskeleton reorganization by phosphorylating LIM kinase and cofilin, leading to increased endothelial cell gaps, elevated vascular permeability, and infiltration of inflammatory factors, thereby exacerbating endothelial damage 36. ROCK also suppresses the expression of tight junction proteins such as zona occludens-1 and occludin, impairing endothelial barrier function 37. Metabolic abnormalities in MetS (such as dyslipidemia and insulin resistance) activate RhoA/ROCK and then directly affect the endothelial cell. Also, in the state of metabolic abnormalities, activated RhoA/ROCK pathway may also damage the endothelial function in CC through other pathways such as promoting the release of inflammatory factors and increasing the inflammatory response in vascular endothelial cells 38, as well as persistently impairing endothelial-dependent vasodilation to reduce endothelial NOS activation by inhibiting the insulin receptor substrate-1/phosphoinositide 3-kinase/protein kinase B pathway 39. Meanwhile, ROCK can enhance the transduction of transforming growth factor-β1/suppressor of mothers against decapentaplegic homolog signaling 40, thereby inducing endothelial-to-mesenchymal transition, increasing fibrosis markers, and structural remodeling of the microvasculature in the CC 41.

### 3.3 Endothelium-derived vasoactive factors

Endothelial cells release various vasoactive factors in response to mechanical forces and neurohumoral mediators, actively regulating vascular tone, inflammatory responses, coagulation balance, and vascular remodeling under both physiological and pathological conditions. The vascular endothelium and cavernosal arteries are a source of vasorelaxing factors such as NO, prostaglandin I2, endothelium-derived hyperpolarizing factor (EDHF), the vasoconstrictor factors such as angiotensin II (Ang II) and endothelin-1 (ET-1), cell adhesion molecules such as Intercellular Cell Adhesion Molecule-1 (ICAM-1) and Vascular Cell Adhesion Molecule-1 (VCAM-1), growth factors such as vascular endothelial growth factor (VEGF), platelet-derived growth factor (PDGF), inflammatory factors, etc. These factors work together to improve CC smooth muscle function, which is essential for maintaining physiological homeostasis in the penile vascular bed in response to changes in blood flow, shear stress, and agonists 42. Bełtowski et al found the impairment of both NO and EDHF in MetS rats, and this impairment probably results in unbalanced sympathetic nervous system stimulation and blood pressure elevation 43. Some clinical studies have shown that excessive production of Ang II, ET-1 are associated with ED, especially those with organic ED 44,45. In normo-weight subjects with MetS, there is a strong association of metabolic parameters with VCAM-1 and Asymmetric Dimethylarginine (ADMA) (a NOS inhibitor) 46, and the aggravation of insulin resistance is often accompanied by abnormal elevations of ICAM-1 and VCAM-1 47, which decrease with the recovery of erectile function and effective treatment in ED patients 48. Also, biochemical measures of endothelial cells activation have been evaluated in patients with ED, and increased ICAM-1, VCAM-1 and ET-1 concentrations may be associated with ED independently of coexisting cardiovascular risk factors and overt vascular damage 49.

### 3.4 Testosterone level

The relationship between endothelial function and testosterone is complex, involving both structural changes and cell signaling pathways 50. Testosterone deficiency leads to endothelial dysfunction, and it may also be related to multiple mechanisms such as regulating endothelial progenitor cells (EPCs) and endothelium-derived vasoactive factors. EPCs serve as fundamental mediators in vascular repair, angiogenesis, and replacing damaged endothelial cells in blood vessels 51. Foresta et al suggested that hypotestosteronemia was related to the reduction of circulating EPCs in young hypogonadotropic hypogonadal men 52, whereas the reduction of EPCs could be reversed by the testosterone replacement through a direct stimulatory effect on the bone marrow 53, and the androgen receptor/VEGF/cyclin A-mediated mechanism may be involved in this process 54. Androgen deficiency regulates endothelial cells by affecting endothelial NOS activity, and then reducing the NO availability 55. Testosterone replacement has potential in reducing ADMA concentrations and increasing NO production in idiopathic hypotrophic hypogonadism 56. Another study by Babcock et al reported that men with low testosterone had higher plasma ET-1, which may be associated with worse brachial artery flow-mediated dilation 57. And the elevation of ET-1 can also activate the RhoA/ROCK pathway in CC then may affect erectile function 58. Testosterone circulating levels tend to a step-ward decrease with ageing as well as the presence of numerous co-morbidities such as MetS 59, and a low level of testosterone may exacerbate obesity, insulin resistance, dyslipidemia, and hypertension 60. Testosterone replacement therapy (TRT) for MetS-associated ED patients can improve MetS conditions (e.g., insulin resistance) and endothelial function 61.

### 3.5 Oxidative stress

The excessive ROS production is strongly associated with endothelial dysfunction and also be recognized as a key contributor to erectile impairment. Uncoupled endothelial NOS, nicotinamide adenine dinucleotide phosphate hydrogen (NADPH) oxidase, xanthine oxidase, and mitochondrial electron transport are the main sources of ROS. When endothelial NOS function is abnormal caused by pathological stimulating factors such as insulin resistance, impaired glucose tolerance and obesity, its catalytic process becomes "uncoupled," leading to the production of superoxide anion (O₂⁻) instead of NO. The uncoupled endothelial NOS generates O₂⁻, which reacts with NO to form ONOO⁻, reducing NO bioavailability while activating pro-inflammatory pathways and exacerbating vascular endothelial damage, marked by diminished vasodilation, elevated vascular resistance, inflammatory activation and reduction of penile blood flow, ultimately impairing erectile function 62. ROS such as hydrogen peroxide serve as signaling molecules, stimulating the action of vasoactive agents like Ang II, ET-1, aldosterone, and prostanoids, while also influencing calcium homeostasis 63. Furthermore, ROS can enhance the expression of pro-inflammatory chemokines and cytokines, leading to the recruitment and activation of immune and inflammatory cells. Clinical evidence showed that there was a lower plasma antioxidant enzyme activity and more biomarkers of oxidative injury in MetS patients compared with healthy subjects 64. MetS, along with overweight, hyperglycemia, etc. is characterized by a proinflammatory state which may result in ED 65. These abnormal inflammatory factors such as tumor necrosis factor-α, interleukin-1β and interleukin-6 may also inhibit the production of testosterone through influencing the steroidogenesis in Leydig cells 66.

### 3.6 Programmed cell death

Programmed cell death such as apoptosis, autophagy, pyroptosis, and ferroptosis plays a significant role in the pathogenesis of vascular ED. Excessive apoptosis reduces endothelial NOS activity as well as interacts with oxidative stress to produce ROS to damage vascular endothelial cells 67. Autophagy maintains vascular endothelial cell homeostasis in the CC by removing damaged organelles, reducing ROS accumulation, stabilizing endothelial NOS, and regulating apoptosis. However, autophagy can also induce excessive apoptosis under pathological conditions 68. Ferroptosis may contribute to the dysfunction of penile vascular endothelial cells through oxidative damage and iron metabolism disorders, and it is likely to occur during the initiation and progression of atherosclerosis 69. Pyroptosis forms pores in the cell membrane via Gasdermin D, releasing pro-inflammatory factors and triggering local inflammation, which impairs endothelial function. Chronic inflammation disrupts endothelial integrity and reduces NO bioavailability, while inflammasomes like NOD-like receptor thermal protein domain associated protein 3 may activate oxidative stress, and finally aggravate vascular damage 70. Although different types of programmed cell death exhibit distinct features, they are interconnected through mutual enhancement, conversion, and suppression. In the state of MetS, oxidized low-density lipoprotein can inhibit autophagic flux by suppressing the sirtuin 1/forkhead box protein O1 pathway to promote apoptosis and adhesion molecule expression in endothelial cells 71. And low androgen levels can induce ferroptosis of endothelial cells in rat penile tissue 72, as well as inhibit erectile function of rats by reducing NOS through pyroptosis of endothelial cells in the CC 73.

## 4. Impacts of MetS-Associated Pathological Conditions on Penile Vascular Endothelium

MetS encompasses pathological conditions mainly including glucose metabolism disorder, hypertension, dyslipidemia and obesity 8,21,22. Each pathological state may damage the vascular endothelium of the CC through multiple potential mechanisms.

### 4.1 Glucose metabolism disorder

There is a clear pathophysiological connection between glucose metabolism disorders (such as DM and insulin resistance) and ED. A meta-analysis found that ED affects more than half of men with the glucose metabolism disorder have a prevalence odds of approximately 3.5 times more than healthy controls 74. ED is considered a key factor connecting sexual dysfunction and cardiovascular issues in DM patients, as endothelial cells are more vulnerable to damage compared to other blood vessel cells 75. Previous research showed reduced expression of genes (e.g., VEGF-A, VEGF-B, VEGF-C) involved in angiogenesis and vascular endothelial function in DM-associated ED compared to healthy men 76. Kurt et al found that DM-associated ED patients have lower flow-mediated dilation, higher ET-1 levels, and elevated ischemia-modified albumin (an early ischemic marker) compared to non-DM-associated ED subjects 77. In addition, reduced serum testosterone levels in men with DM are an independent factor of endothelial dysfunction, contributing to vascular disease and ED 78, and evidence also showed a decrease in circulating EPC correlates with penile endothelial dysfunction and testosterone levels in DM-associated ED 79.

To further investigate how abnormal glucose metabolism induces vascular endothelial damage in the penis, Felten et al showed that endothelium-dependent vasodilation in the rat penis can be reversed by high levels of L-arginine 80. Also, elevated binding of ligands to the potent vasoconstrictor, ET-1 and endothelin A and endothelin B receptors have been noted in rat and rabbit penile tissues 81. In diabetic rodent penile tissue, altered expression of angiogenic mediators like VEGF and Ang-1, which disrupt intracellular signaling pathways 82,83. It is well acknowledged, VEGF expression is reduced in diabetic CC, affecting VEGF-mediated signaling, decreasing endothelial NOS activation, and reducing endothelial cell viability 84. Similarly, Ang-1 therapy administered intracorporeally boosted endothelial NOS activation in DM rat, enhancing endothelial content and ameliorating ED 83. In addition to the endothelium-derived relaxing factors and endothelium-derived contracting factor, inflammatory factors also play a significant role in influencing the vascular endothelium of the penis. From the evidence of Mao et al, interleukin-6, tumor necrosis factor-α, and interleukin-1β in serum levels of DM-associated ED rats were increased, while interleukin-10 and interleukin-4 were decreased 85. In addition, the change of inflammatory factors often followed with oxidative stress and programmed cell death 85.

Limited literature exists on oxidative stress marker changes in penile tissue from DM patients. In DM rats, these changes mirror those in other vascular areas, including high malondialdehyde levels and low glutathione concentrations 86. Confocal microscopy imaging of DM penile tissue showed elevated superoxide production in the vascular endothelium, and ROS may compromise NO bioavailability, which contributes to endothelial dysfunction and results in long-term vascular impairment in DM penile tissues. Studies have also demonstrated that the use of ROS scavengers can significantly improve penile vascular endothelial defects in streptozotocin-induced DM rats 87. Meanwhile, oxidative stress can trigger programmed cell death. Increased oxidative stress elevates cell apoptosis while reducing endothelial content, ultimately impairing penile hemodynamics and erectile function in animal models 88. According to Li et al, the proportion of endothelial cell pyroptosis (24.4 ± 3.69%), endothelial cell apoptosis (22.13 ± 2.43%), total cell pyroptosis (14.75 ± 0.93%), and total apoptosis (14.82 ± 1.08%) in the CC of the DM rats were significantly greater than those in the normoglycemic rats (*P* < 0.01) 89. In fact, apoptosis occurring in the endothelial cells of penile blood vessels has also been confirmed by the clinical evidence 90. However, there is few evidence regarding autophagy, ferroptosis and pyroptosis occurring in the vascular endothelial cells of the CC in patients with DM-associated ED. Li et al observed that endothelial cell pyroptosis is the dominant form of cell death in the initial phases of DMED development 89, and suppressing NOD-like receptor thermal protein domain associated protein 3 expression can significantly reduce pyroptotic activity in diabetic penile tissue of rats, enhancing the vascular endothelial function 91. Xin et al found that the nuclear factor erythroid 2-related factor 2-heme oxygenase-1/glutathione peroxidase 4 axis in DM-associated ED rats was inhibited, leading to ferroptosis and oxidative stress, which impaired endothelial/smooth muscle cell function and further aggravated cavernous fibrosis 92. Another study demonstrated that the NO/cGMP pathway was inhibited in the DM-associated ED rats, accompanied by the occurrence of autophagy and apoptosis, as well as the high expression of mammalian target of rapamycin in the penis 93.

RhoA/ROCK activity is elevated in CC of DM-associated ED rats 94. RhoA/ROCK can promote advanced glycation end products (AGEs) in high glucose environment 95. AGEs are irreversible compounds formed when sugars react with proteins, lipids, or nucleic acids without enzyme control (glycation) 96. Research indicated that chronic hyperglycemia stimulates AGEs production, which disrupt NO generation in endothelial cells by blocking endothelial NOS phosphorylation 97. AGEs also contribute to the generation of ROS, and affect the expression of endothelial growth factors to damage endothelial function in penis 98.

### 4.2 Hypertension

Epidemiological data showed that ED affects more than 30% of individuals with hypertension, compared to a prevalence rate of about 9.6% in the overall population 99. Jensen et al observed that the main reason for ED among hypertensive patients were penile impairment (found in 89%), probably due to atherosclerosis 100. Hypertension may compromise penile blood flow through vascular damage affecting the pudendal arteries as well as penile blood vessels 101. Elevated blood pressure causes morphological changes in the penile vasculature and ED, and hypertension-induced remodeling, which is characterised by fibre derangement and mild cytoplasmic inflammatory may also substantially impair arterial inflow to the penis 102. Hypertension promotes the release of vasoactive compounds, stimulates endothelial cell proliferation, and causes damage to smooth muscle cells, Schwann cells, as well as increased vascular intimal permeability 103. Specifically, common effects of hypertension on the penile vascular endothelium involve in disorders of NO/cGMP pathway, RhoA/ROCK pathway, endothelium-derived vasoactive factors, oxidative stress, and programmed cell death.

The role of NO and other possible mediators in endothelial dysfunction of hypertensive rats 104,105. In spontaneously hypertensive rats, endothelial-mediated relaxation of CC strips in response to acetylcholine was significantly impaired, suggesting a defect in endothelium-dependent reactivity and a corresponding reduction in NO 106. Clinical evidence showed that endothelial function of hypertensive patients has an inverse relationship with ADMA (a competitive inhibitor of endothelial NOS) 107. Endothelial NOS was also significantly decreased in the hypertensive animal model 108. Therefore, impairment of NO/cGMP pathway in hypertensive rats may explain reduced efficacy of phosphodiesterase-5 inhibitor (PDE5I) as observed in the previous experiments 109. In addition, other relevant pathologic mechanisms may also influence hypertension-associated endothelial injury in penile vasculature by acting directly or indirectly on the NO/cGMP pathway.

ED may arise from an impaired response to vasoconstrictive stimuli like Ang II and ET-1. In fact, elevated Ang II and ET-1 levels in systemic and cavernosal blood have been observed in hypertensive men with ED 110,111. As the strongest vasoconstrictor, ET-1 may also constrict the internal pudendal artery (the main blood vessel supplying the penis), exacerbating localized blood flow disturbances in the penis. Hypertension is closely linked to Ang II, a potent vasoconstrictor that influences its onset and chronic persistence. Ang II regulates aldosterone production in the adrenal gland 112. It also influences cardiovascular homeostasis by activating the Ang II type 1 receptor, mainly found in cardiovascular cells 113. Excessive activation of the Ang II type 1 receptor induces NADPH oxidase, which is a major generator of ROS in vascular and CC, and elevated ROS level may diminish NO bioavailability. Clinical evidence demonstrated that increased ROS production and decreased NO bioavailability were in hypertensive patients 114. Elevated levels of spongy lipid peroxidation in the CC of hypertensive rats 115. High levels of ROS may diminish the availability of NO, either via peroxynitrite production or through other mechanisms. In addition, Ang II and aldosterone can interact and jointly contribute to the progression of target organ damage by activating the RhoA/ROCK pathway 116. Similarly, the overactivation of ET-1 has also been identified as a significant pathway for the activation of the RhoA/ROCK signaling 117. The protein expression of ROCK1 and ROCK2 were significantly higher in hypertension-associated ED rats than that in the normal control rats, with a reduction of endothelial NOS, cGMP, and an elevation of norepinephrine-induced hyper-contractions, and acetylcholine-induced hypo-relaxations in the penile tissue 118.

Penile vascular endothelial injury in hypertension-associated ED rats is also related to the programmed cell death. Luan et al found endothelial dysfunction in hypertension-associated ED rats, with the increased expression of the NOD-like receptor thermal protein domain associated protein 3 inflammasome, caspase-1, gasdermin-D, and the pro-inflammatory cytokines interleukin-1β and interleukin-18 in the CC, supporting the involvement of pyroptosis in vascular endothelial injury related to hypertension-associated ED 119. In addition to pyroptosis, when hypertensive state exists alone in rats, caspase 3-mediated apoptosis, beclin-1/LC3-II-mediated autophagy, may also decrease the expression of NO and endothelial NOS in the penis, and finally affect the penile vascular endothelium through multiple pathways 120.

### 4.3 Dyslipidemia

There is a close association between dyslipidemia and penile vascular endothelial dysfunction. In a study of 154 men with ED, 74% had a low-density lipoprotein cholesterol level greater than 120 mg/dl (3.1 mmol/l) 121. From the results of Wei et al, every mmol/liter of increase in total cholesterol was associated with 1.32 times the risk of ED (95% confidence interval 1.04-1.68), while every mmol/liter of increase in high density lipoprotein cholesterol was associated with 0.38 times the risk (95% confidence interval 0.18-0.80) 122. Similarly, an elevated triglyceride/high-density lipoprotein ratio emerged as an independent risk factor for ED, with vascular endothelial dysfunction serving as the probable underlying cause 123. Dyslipidemia and ED were significantly related in older men, possibly because atherosclerotic damage takes longer to develop 124. Due to the limited clinical data, some published animal experiments have shown that hyperlipidemia-associated ED rats may exhibit reduced endothelial cell numbers, downregulated cell-to-cell junctions, increased CC fibrosis, and a lower smooth muscle-to-collagen ratio 125. Gholami et al also found a decrease in the content of endothelial cells in the CC of the high-cholesterol rat model 126. Meanwhile, electron microscopy in cholesterol plus phosphate buffered saline treated rats showed denuded endothelial lining of the sinusoids covered by numerous platelets in the CC 126. From the previous evidence, in dyslipidemia-related ED, angiogenesis is disrupted due to (1) endothelial NOS downregulation and NO depletion, (2) superoxide-mediated NO degradation, and (3) oxidized low-density lipoprotein/lysophosphatidylcholine-induced inhibition of endothelial migration—key processes driven by arterial wall atherogenesis 127. Moreover, the pathological mechanisms of these endothelial injuries seem to be attributed to disorders of the NO/cGMP pathway, abnormal endothelium-derived vasoactive factors, oxidative stress, etc.

In apolipoprotein E knockout mice aged 30-35 weeks, researchers observed significant impairment of NO-dependent endothelial relaxation in aortic tissue 128,129. Interestingly, although NOS enzymatic activity remained normal during initial disease progression 130, the bioavailability of NO was already reduced 129. Another study by Xie et al showed that the high-cholesterol diet induced time-dependent alterations in endothelial-dependent and -independent vascular responses, endothelial cell density, smooth muscle-to-collagen ratio, selective phospho-endothelial NOS-Ser1177 expression, and cGMP levels in the penis of mouse model 131. Among them, the endothelium-dependent relaxation function of mice on a high-cholesterol diet was impaired at the 8th and 12th weeks. Additionally, the ratio of phosphorylation of endothelial NOS at Ser1177/total endothelial NOS decreased to 46% at the 12th week, while the cGMP level significantly dropped at the 12th week. This further indicated that a high-cholesterol diet may damage vascular endothelium by affecting the NO/cGMP pathway, thereby reducing erectile function 131.

In rabbits fed a 0.5% high-cholesterol diet, VEGF receptor-2 decreased in the CC, while VEGF increased initially but declined later 132, another study by Ryu et al also showed that in rats on a 4% high-cholesterol diet for 3 months, VEGF and VEGF receptor-2 were downregulated in the CC 133. VEGF is an angiogenic growth factor that participates in promoting angiogenesis and vascular permeability in both physiological and pathological processes. In vascular ED, it mainly promotes angiogenesis, improves endothelial function, and enhances penile blood perfusion 134. Similarly, intracavernous injection of VEGF seems to be able to improve the endothelial injury in the CC caused by hyperlipidemia in the rabbit model 135.

Oxidative stress is also involved in the endothelial injury of the CC vessels related to dyslipidemia. The CC of animals subjected to a high-cholesterol diet demonstrated increased ROS 136. NADPH oxidase activation acts as a key contributor to oxidative stress, resulting in endothelial NOS disorder and exacerbating endothelial impairment in hypercholesterolemia-associated ED 136. In the rat model of hyperlipidemia-associated ED, oxidative stress responses occur in the endothelial cells of the CC, accompanied by an increase in the apoptotic index 137. In addition, oxidized low-density lipoprotein plays a significant role in hyperlipidemia-associated ED, as elevated levels have been detected in the human penile tissue 138. Within the vascular system, oxidized low-density lipoprotein enhances superoxide generation through multiple pathways, including the induction of uncoupled endothelial NOS, activation of NADPH oxidase and xanthine oxidase enzymes, and disruption of mitochondrial electron transport chain function 139. However, the role of oxidized low-density lipoprotein in penile vessels and how it affects endothelial cells remains to be further elucidated.

### 4.4 Obesity

Obesity is a major global health concern and often associates with ED. Men with abdominal obesity may have an elevated ED risk, and the severity of obesity is also directly proportional to the severity of ED 140. In a cross-sectional study based on National Health and Nutrition Examination Surveys, higher visceral adiposity index is independently related to ED risk 141, The visceral adiposity index can clearly reveal the influence of waist circumference, body mass index, triglyceride, etc., and is considered more practical in assessing the impact of obesity on ED patients 142. However, for metabolically healthy patients who meet obesity criteria, the impact on erectile function may be smaller due to the absence of organic damage. As Moura et al have verified, metabolically unhealthy obese individuals had lower mean peak systolic velocity (28.1 cm/s vs. 36.9 cm/s) and IIEF-5 than metabolically healthy obese individuals, while there were no significant differences in IIEF-5 scores, mean peak systolic velocity, or hypogonadism prevalence found between metabolically healthy obese and non-obese patients 143. Similarly, in the experiment by Odom et al, mice fed a high-fat diet for 12 weeks developed obesity characteristics, and interestingly, endothelium-dependent and nondependent diastolic function was unchanged in both systemic and penile vasculature, and the 12-week high-fat diet did not affect penile neurotransmitter-mediated diastolic function 144. We speculate that these normal obese subjects may not have insulin resistance or hypogonadism or that this healthy metabolic state of obesity does not cause significant organic damage in a short period of time. In fact, metabolically healthy obese individuals are likely to become metabolically unhealthy in subsequent years with a high probability 145. A clinical trial reported obesity without insulin resistance preserved endothelial function while insulin-resistant obese individuals had endothelial dysfunction 146. Indeed, insulin resistance is an independent risk factor for ED in young men as clinical evidence confirmed 147. Also, obesity disrupts the hypothalamic-pituitary-gonadal axis, causing hypogonadism and testosterone deficiency, while adipose-mediated aromatization of testosterone to estrogen exacerbates hormonal dysregulation. Corona et al showed that among 2435 ED patients, obesity-related comorbidities with low testosterone levels correlated more strongly with impaired penile blood flow than obesity alone 148. Although there is debate about whether obesity causes ED, but evidence showed that obesity-induced metabolic abnormalities do lead to ED. And these obesity-associated ED studies also emphasized the importance of penile vascular endothelial function 146,149, which were related to NO/cGMP pathway, low testosterone levels, adipokine dysregulation, oxidative stress, etc.

In obese animals, ED is associated with decreased NO bioavailability in erectile tissue. On the one hand, the low testosterone level associated with obesity affects the function of penile vascular endothelium and reduces the activity of endothelial NOS through multiple pathways. On the other hand, insulin resistance also mediates the connection between obesity and NO deficiency 150. The insulin induces vasodilation by increasing the expression of endothelial NOS and NO production through the activation of phosphoinositide 3-kinase/protein kinase B pathways 151. Elevated levels of ROS can lead to a decrease in NO levels and impaired endothelial cell function. Current evidence suggest that a significant portion of ROS in the penile vascular endothelium of obese rats with ED is derived from adipokine dysregulation 149. Adipokines, which are cytokines secreted by adipose tissue, play crucial roles in regulating metabolic functions, including energy metabolism, inflammatory responses, and vascular homeostasis. Dysregulated adipokines disrupts normal adipokine signaling, promoting oxidative stress through ROS generation and contributing to endothelial dysfunction 152. In the state of obesity, pro-inflammatory adipokines (such as leptin, resistin, etc.) directly promote inflammatory responses, and anti-inflammatory adipokines (such as adiponectin) are inhibited. A decrease in adiponectin levels observed in obese patients is thought to contribute to insulin resistance 153. Adiponectin also improves endothelial function, reduces endothelial cell apoptosis, and decreases the risk of atherosclerosis by reducing the expression of adhesion molecules in blood vessels, the formation of foam cells, and the proliferation of vascular smooth muscle cells 154. This is also the reason why obese patients with low levels of adiponectin are more prone to vascular endothelial dysfunction 155. Meanwhile, hypoxia and cell death caused by the expansion due to adipose tissue accumulation further recruit macrophages and promote the release of tumor necrosis factor-α, interleukin-6, and interleukin-1β, which induce inflammatory gene transcript in endothelial cells and suppresses endothelial NOS expression 156. Clinical evidence showed that inflammatory cytokines in the plasma of obese individuals are related to body mass index 157. These highly expressed pro-inflammatory factors may cause inflammatory responses and insulin resistance, and high levels of tumor necrosis factor-α are associated with the occurrence of ED 158. In animal models, tumor necrosis factor-α perfusion of the CC showed weakened non-adrenergic non-cholinergic nerve-mediated relaxation, and the expression of endothelial NOS was inhibited 159.

## 5. Management and Potential Drug Targets for Penile Vascular Endothelial Dysfunction in Patients with Metabolic Syndrome

Currently, a healthy lifestyle is the preferred treatment option for organic ED according to sexual medicine experts 160. Firstly, evidence from systematic reviews has shown that aerobic training can significantly enhance male erectile function 161, which may be related to mechanisms such as exercise reducing oxidative stress, increasing the production of lactic acid, NO, and cortisol, and raising total testosterone levels 162. Preclinical studies in a rabbit model of MetS-associated hyperglycemia have demonstrated that endurance exercise alone can restore the normal function of the hypothalamic-pituitary-gonadal axis 163. Secondly, ED caused by smoking is mainly related to endothelial damage, reduced NO, and oxidative stress. Therefore, quitting smoking helps restore erectile function 164. Moreover, a healthy diet, including one rich in fruits and vegetables and low in fat, has been shown to be associated with less ED in the general population 165, and the potential of plant-based diets in MetS-associated ED was also highlighted 166. Finally, limiting alcohol intake, controlling weight, maintaining regular sleep, and avoiding emotional stress are all crucial for MetS-associated ED.

MetS-associated ED involves the management of multiple diseases. For individuals with insulin resistance and DM, the viewpoint of Corona et al indicates that metformin, glucagon-like peptide-1 agonist, and sodium-glucose cotransporter-2 inhibitor are more effective in improving ED 167. Metformin is recommended as the first-line treatment for type 2 DM, as it can improve endothelial dysfunction by enhancing endothelium-dependent vasodilation and reducing sympathetic overactivity, ultimately restoring erectile responses in the CC of DM animal models through the NO/cGMP pathway 168. Vignozzi et al also showed that long-term use of metformin can restore erectile function in MetS animal models by increasing adenosine and NO signaling 169. For individuals with a higher body mass index, factors such as lipid metabolism disorders and decreased testosterone levels have a more significant impact on erectile function. Glucagon-like peptide-1 receptor agonists improve erectile function by reducing weight and regulating lipid metabolism, and show more significant improvement in erectile function compared to metformin 170. Other antidiabetic drugs, such as thiazolidinediones and α-glucosidase inhibitors, show promising mechanisms but lack strong clinical validation for their effects on ED 170. The impact of antihypertensive drugs represented by β-blockers on ED has been confirmed. β-blockers may cause ED by reducing perfusion pressure, directly acting on penile and vascular smooth muscle cells, or reducing testosterone levels, which also decreases patients' treatment compliance 171. However, evidence from meta-analyses indicated that nebivolol can significantly reduce the incidence and progression risk of ED 172. As a third-generation β-blocker, nebivolol blocks α-adrenergic receptors and induces the release of NO, showing significant advantages in reversing ED caused by first- and second-generation β-blockers. Additionally, inhibiting Ang II has a positive impact on endothelial function in patients with hypertension and MetS 173, and patients with hypertension-associated ED can benefit from the treatment with angiotensin-converting enzyme inhibitors and angiotensin receptor blockers 174. In animal experiments, angiotensin receptor blockers can reduce ROS generation in penile tissues of aged rats and hypercholesterolemic mice and enhance NO production 175,176. For patients with dyslipidemia-associated ED, statins not only lower cholesterol but also show the effect of enhancing the activity of endothelial NOS 177, and have potential in improving MetS-associated ED. Park et al reported that the target of statins in improving ED lies in antioxidation and inhibition of the RhoA/ROCK signaling pathway 178. However, these evidences seem insufficient and further in-depth research is still needed.

Vascular endothelial dysfunction in MetS-associated ED involves multiple mechanisms discussed in our review (Table 1). Due to individual differences and underlying diseases, individualized therapy should be implemented while providing necessary treatment for the underlying diseases, focusing on endothelial-related therapeutic targets. It is well known that PDE5I is the first-line treatment recommended by guidelines and is effective for various organic ED, including MetS-associated ED. However, the main target of PDE5I is cGMP, which is hydrolyzed to inactive 5'-guanylic acid. Although PDE5I may have positive effects on improving NO and endothelial NOS and inhibiting ROCK 179, but more clinical evidence is still needed to support it. For MetS-associated ED patients, PDE5I, which is often well-tolerated and safe, should also take into account the high failure rate of PDE5I monotherapy for MetS-associated ED 180. Also, all the different formulations of organic nitrates are considered to increase NO levels, which in turn increases cGMP levels, and the combined application with PDE5I often leads to hypotension and is not recommended in clinical practice. Testosterone plays a significant role in MetS-associated ED. Testosterone replacement therapy including the use of oral formulations of testosterone undecanoate, has significantly increased testosterone levels and erectile function in patients with low androgen levels, and it has long-term safety 181. However, patients may still face the problem of hypogonadism after discontinuation of the drug. To overcome this issue, many researchers have attempted to combine daily injections of testosterone undecanoate with PDE5I, which has further improved the sustained therapeutic effect 182 and is recommended for ED patients with obesity and type 2 DM 183. At the same time, for patients with low testosterone levels, TRT also shows potential in improving total cholesterol levels 184. Nevertheless, the current situation of androgen abuse should also be taken seriously. For instance, TRT is not recommended in case of ED with normal testosterone level, as well as in men seeking for reproductive potential, due to inhibition of the spermatogenesis 185. The extensive research on RhoA/ROCK pathway in endothelial dysfunction related to MetS-associated ED has made it a reasonable drug target. Sopko et al reviewed the physiological and pathological mechanisms of the RhoA/ROCK pathway in penile vascular endothelial dysfunction and summarized the possible treatment directions 186. Although Lasker et al found that selective ROCK inhibitor can improve erectile function in rats with nerve injury and independently of the NO/cGMP pathway 187. There is still a long way to go for the application of the ROCK inhibitor in patients troubled by MetS-associated ED, including the complexity of MetS etiology and the adverse reactions such as systemic blood pressure reduction after use of ROCK inhibitors 186.

Regenerative therapy in penile vascular endothelium may cure MetS-associated ED by enhancing vascular endothelial growth factor, regulating the NO/cGMP pathway, and controlling programmed cell death. Evidence indicated that low-intensity extracorporeal shock wave therapy can cause minor damage to vascular endothelial cells, stimulating the release of VEGF, promoting new blood vessel formation, and restoring penile blood flow 188, and has been widely applied in clinical practice 189. The potential of stem cell therapy in improving MetS-associated endothelial dysfunction should not be overlooked. Liu et al's research demonstrated that erectile function recovery in DM rats was observed following the administration of VEGF-secreting adipose derived stem cells, which enhanced endothelial function and increased smooth muscle and pericyte levels through VEGF action 190. Human urine-derived stem cells isolated from the urine of healthy adult males, when injected into the CC of DM-associated ED rats, could increase autophagic activity in CC endothelial cells, thereby improving vascular endothelial function 191. Additionally, extracellular vesicles from human urine-derived stem cells can promote endothelial cell proliferation and angiogenesis, increase endothelial NOS content in the CC. RNA sequencing analysis results also indicate their potential in enhancing vascular endothelial integrity 192. Due to the high cost, the lack of long-term risk assessment, individual differences in therapeutic effects, the absence of standardized dosing regimens, and certain ethical controversies, the stem cell therapy has not yet been included in the routine recommended treatment options for ED.

In clinical practice, the efficacy of using antioxidants alone to treat ED is limited, and they are often used as complementary and alternative therapies in ED patients. By combining with PDE5I, they can exert therapeutic advantages for vascular ED 193. The impact of antioxidants on ED has been studied in various animal models, such as vitamin E, melatonin, and α-lipoic acid, which have been confirmed to improve penile vascular lesions related to MetS 194. Our team has summarized the mechanism of action of medicinal plants on DM-associated ED in previous review 195. Many medicinal plants have the advantage of multi-target regulation of DM-associated ED. They can not only regulate oxidative stress, inhibit malondialdehyde and ROS in CC, and activate the NO/cGMP pathway, but also play an important role in endothelial dysfunction caused by programmed cell death 195. Therefore, in some East Asian countries, many researchers have mixed several medicinal plants to make traditional Chinese medicine prescriptions, which also have potential in the treatment of MetS-associated ED as well as other male reproductive diseases 196,197. In addition, as we mentioned, plant-based diets are receiving increasing attention. By evaluating the impact of dietary antioxidants and plant extracts on penile vascular endothelial damage related to MetS, researchers can develop effective antioxidant-based treatment strategies that are easier to implement in daily life and facilitate patient management.

## 6. Perspectives and Future Directions

**Figure 2:**
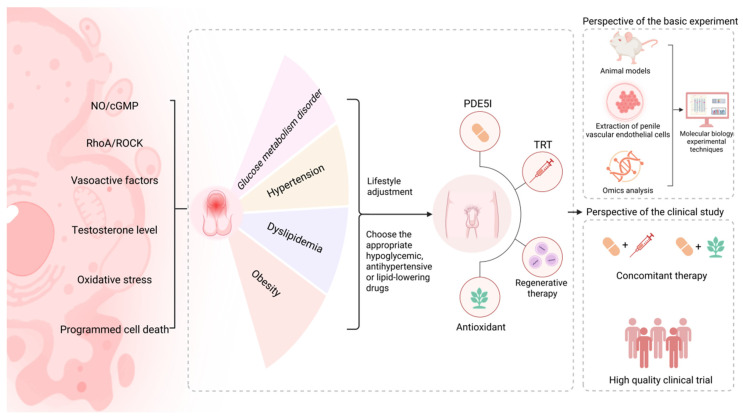
Common therapies for penile vascular endothelial injury associated with MetS-associated ED and future research directions. Abbreviations: NO, nitric oxide; cGMP, cyclic guanosine monophosphate; RhoA, ras homolog family member A; ROCK, rho-associated coiled-coil containing protein kinase; PDE5I, phosphodiesterase-5 inhibitor; TRT, testosterone replacement therapy. Created in BioRender. Wang, H. (2025) https://BioRender.com/fbi4hq5.

We reviewed the multiple mechanisms of penile vascular endothelial injury related to ED in MetS and briefly analyzed the current treatment progress (Figure 2). Combining the existing evidence, the treatment of CC vascular endothelial injury based on the RhoA/ROCK pathway still faces numerous challenges. Therefore, the NO/cGMP pathway remains a key focus for future research, serves as the core target for treating CC vascular endothelial injury in MetS-associated ED. Although modulating endothelium-derived vasoactive factors, antioxidation and regulating programmed cell death are beneficial for improving CC endothelial dysfunction related to MetS, limited literature evidence, low bioavailability, lack of cell specificity and low clinical efficacy as well as other factors have restricted the drug development based on these targets. The existing MetS-associated animal models induced by high-fat diets are mainly rodent models, including (diabetic ED rats, hyperlipidemic ED rats, etc.), which may only lead to obesity or insulin resistance, but lack key manifestations such as hypertension or inflammation. In addition, animals with simple metabolic abnormalities are difficult to show significant ED in a short time and may require additional interventions (such as vascular injury, drug induction), which further increases the difficulty and cost of modeling. Due to ethical limitations on extracting endothelial cells from human CC, human umbilical vein endothelial cells are commonly used because of their easy accessibility and ease of culture. However, they differ from microvascular endothelial cells in the CC in terms of gene expression, functional characteristics, and response to androgens, making it difficult to fully simulate the local pathological and physiological processes of the penis. The extraction of endothelial cells from animal models reduces ethical risks and enables controlled mechanism exploration. This also means that on the basis of optimizing the MetS animal model, the extraction technology should be continuously improved to ensure the activity and purity of penile vascular cells. In addition, by screening differentially expressed genes (such as endothelial NOS and inflammatory factors) through transcriptomics, validating key proteins (such as VEGF and ICAM-1) through proteomics, and analyzing small molecule metabolites (such as NO and oxidative stress markers) through metabolomics, a molecular network of endothelial injury can be jointly constructed 33. This will facilitate the discovery of biomarkers and therapeutic targets for endothelial injury related to MetS-associated ED, and provide personalized treatment strategies.

With the in-depth research on combined therapy for MetS-associated ED, simultaneous treatment of metabolic control and ED is recommended. Clinical physicians should monitor the metabolic-related indicators and the patients' own conditions in a timely manner, and guide the basic medication with necessary lifestyle guidance. This also means the participation of multiple disciplines, including the department of sexual medicine, urology, nutrition, cardiology, and endocrinology, to provide effective and comprehensive guidance for personalized management. In terms of improving the quality of sexual life, concomitant therapy (including the application of PDE5I combined with TRT or PDE5I combined with antioxidant therapy, etc.) is still necessary 193,196, which involves multi-target regulation of ED related to MetS. We expect more high-level clinical evidence to confirm the efficacy and safety of concomitant therapy for MetS-associated ED patients, and also expect the clinical application value of regenerative therapy, which may benefit those patients who are ineffective with PDE5I and unwilling to receive the penile prosthesis implantation.

## 7. Conclusion

MetS-associated ED is a refractory ED. Due to the multiple pathological features included in MetS, it involves various different mechanisms. Existing studies have clarified that MetS causes a decline in erectile quality and sexual life satisfaction in patients by affecting penile vascular endothelial function. NO/cGMP pathway, RhoA/ROCK pathway, endothelium-derived vasoactive factors, testosterone level, oxidative stress, programmed cell death, etc. may be involved in the penile vascular endothelial dysfunction of MetS-associated ED, and are reflected in the main pathological features of MetS such as glucose metabolism disorder, hypertension, dyslipidemia, and obesity. Currently, the drug targets for treating penile vascular endothelial injury in MetS-associated ED mainly focus on the NO/cGMP pathway. Patients can benefit from lifestyle adjustments and the rational application of hypoglycemic drugs, antihypertensive drugs, or lipid-lowering drugs. At the same time, the application of combined therapy also has potential in the management of MetS-associated ED.

## Figures and Tables

**Table 1:** MetS pathological manifestations and their main relevant mechanisms

MetS pathological manifestations	Main mechanisms in endothelial dysfunction of ED	Ref.
glucose metabolism disorder	NO/cGMP pathway RhoA/ROCK pathway endothelium-derived vasoactive factors sex hormone levels oxidative stress programmed cell death	80-82,87,90,94
hypertension	NO/cGMP pathway RhoA/ROCK pathway endothelium-derived vasoactive factors oxidative stress programmed cell death	106,110,114,116,118,119
dyslipidemia	NO/cGMP pathway endothelium-derived vasoactive factors oxidative stress	131,133,136,137
obesity	NO/cGMP pathway sex hormone levels oxidative stress	148-150

**Abbreviation:** ED, erectile dysfunction; MetS, metabolic syndrome; NO, nitric oxide; cGMP, cyclic guanosine monophosphate; RhoA, ras homolog family member A; ROCK, rho-associated coiled-coil containing protein kinase
